# Detection and Quantification of the Capsular Polysaccharide of Burkholderia pseudomallei in Serum and Urine Samples from Melioidosis Patients

**DOI:** 10.1128/spectrum.00765-22

**Published:** 2022-08-04

**Authors:** Haley L. DeMers, Teerapat Nualnoi, Peter Thorkildson, Derrick Hau, Emily E. Hannah, Heather R. Green, Sujata G. Pandit, Marcellene A. Gates-Hollingsworth, Latsaniphone Boutthasavong, Manophab Luangraj, Kate L. Woods, David Dance, David P. AuCoin

**Affiliations:** a Department of Microbiology and Immunology, University of Nevada, Reno, School of Medicine, Reno, Nevada, USA; b Lao-Oxford-Mahosot Hospital Wellcome Trust Research Unit, Microbiology Laboratory, Mahosot Hospital, Vientiane, Lao People’s Democratic Republic; c Centre for Tropical Medicine and Global Health, University of Oxford, Oxford, United Kingdom; d Faculty of Infectious and Tropical Diseases, London School of Hygiene and Tropical Medicine, London, United Kingdom; Johns Hopkins Hospital

**Keywords:** *Burkholderia pseudomallei*, lateral flow immunoassay, melioidosis, rapid diagnostic test

## Abstract

Burkholderia pseudomallei is the causative agent of melioidosis, a life-threatening disease common in Southeast Asia and northern Australia. Melioidosis often presents with nonspecific symptoms and has a fatality rate of upwards of 70% when left untreated. The gold standard for diagnosis is culturing B. pseudomallei from patient samples. Bacterial culture, however, can take up to 7 days, and its sensitivity is poor, at roughly 60%. The successful administration of appropriate antibiotics is reliant on rapid and accurate diagnosis. Hence, there is a genuine need for new diagnostics for this deadly pathogen. The Active Melioidosis Detect (AMD) lateral flow immunoassay (LFI) detects the capsular polysaccharide (CPS) of B. pseudomallei. The assay is designed for use on various clinical samples, including serum and urine; however, there are limited data to support which clinical matrices are the best candidates for detecting CPS. In this study, concentrations of CPS in paired serum and urine samples from melioidosis patients were determined using a quantitative antigen capture enzyme-linked immunosorbent assay. In parallel, samples were tested with the AMD LFI, and the results of the two immunoassays were compared. Additionally, centrifugal concentration was performed on a subset of urine samples to determine if this method may improve detection when CPS levels are initially low or undetectable. The results indicate that while CPS levels varied within the two matrices, there tended to be higher concentrations in urine. The AMD LFI detected CPS in 40.5% of urine samples, compared to 6.5% of serum samples, suggesting that urine is a preferable matrix for point-of-care diagnostic assays.

**IMPORTANCE** Melioidosis is very challenging to diagnose. There is a clear need for a point-of-care assay for the detection of B. pseudomallei antigen directly from patient samples. The Active Melioidosis Detect lateral flow immunoassay detects the capsular polysaccharide (CPS) of B. pseudomallei and is designed for use on various clinical samples, including serum and urine. However, there are limited data regarding which clinical matrix is preferable for the detection of CPS. This study addresses this question by examining quantitative CPS levels in paired serum and urine samples and relating them to clinical parameters. Additionally, centrifugal concentration was performed on a subset of urine samples to determine whether this might enable the detection of CPS in samples in which it was initially present at low or undetectable levels. These results provide valuable insights into the detection of CPS in patients with melioidosis and suggest potential ways forward in the diagnosis and treatment of this challenging disease.

## INTRODUCTION

Burkholderia pseudomallei is a Gram-negative pathogenic bacterium responsible for melioidosis, a life-threatening infection with clinical manifestations that can range from a skin abscess to severe pneumonia and septicemia. This soil-dwelling saprophytic bacterium is widely endemic in the tropics, particularly in northern Australia and Southeast Asia (1, 2). While the true geographic distribution of the bacterium and the global burden of the disease are not fully understood, modeling has suggested that there may be 165,000 cases of melioidosis worldwide, resulting in approximately 89,000 fatalities annually (3).

Cases of melioidosis are highly underreported, in part due to nonspecific symptoms that can make clinical diagnosis challenging and also difficulties with laboratory confirmation of the diagnosis (3–5). Patients with melioidosis often present with an undifferentiated fever, but if left untreated, the disease can progress to severe septicemia and multiorgan dysfunction and abscesses (6). B. pseudomallei is intrinsically resistant to many antibiotics, including penicillin and first- and second-generation cephalosporins (7). Notably, the mortality rate of melioidosis has been reported to be as high as 70% in patients who did not receive the correct antibiotic treatment (3). Consequently, accurate diagnosis is critical for early and effective treatment.

The current gold standard for diagnosing melioidosis relies on culturing B. pseudomallei from a patient sample. This process takes a minimum of 48 to 72 h and often requires up to 7 days to yield results (8–10). In addition to a long time to a result that subsequently delays informed treatment decisions, the sensitivity of culture is low, at approximately 60% (11). Despite its shortcomings, culture has remained the gold standard, primarily due to the lack of a better alternative. In recent years, a melioidosis rapid diagnostic test (RDT) with the potential to expand the available tools for the challenging task of diagnosing melioidosis has been developed. The Active Melioidosis Detect (AMD) lateral flow immunoassay (LFI) is designed to detect the capsular polysaccharide (CPS) of B. pseudomallei in various types of patient samples (e.g., blood, urine, and pus) and also in turbid blood cultures (2, 12–15). A key virulence factor, B. pseudomallei CPS is an unbranched homopolymer of 1,3-linked 2-*O*-acetyl-6-deoxy-β-d-*manno*-heptopyranose residues that is shed by the bacterium and known to circulate during infection (16–18).

Data on the concentrations of CPS in patient samples are still fairly limited, and more needs to be known about the levels of this circulating antigen in different sample matrices collected from melioidosis patients. The present study was performed to expand on current knowledge by examining paired serum and urine samples collected from melioidosis patients in the Lao People’s Democratic Republic (Laos). Patient samples were analyzed by a quantitative antigen capture enzyme-linked immunosorbent assay (ELISA) to determine CPS concentrations and were tested in parallel on the AMD LFI. Additionally, a subset of urine samples was further assayed and analyzed to determine whether concentration by centrifugal filtration may improve detection in otherwise CPS-negative or low-CPS samples.

## RESULTS

A total of 73 samples (31 serum and 42 urine samples) were obtained from 34 patients with culture-positive melioidosis and 1 patient whose cultures were negative but whose initial diagnosis of melioidosis was based on a weakly positive urine AMD LFI result. All 34 culture-confirmed patients had a set of blood cultures taken on the same date as when the serum/urine samples included in this analysis were taken; 20/34 of these patients were bacteremic with B. pseudomallei.

### Quantitation of CPS in patient samples by an antigen capture ELISA.

A CPS antigen capture ELISA was used to determine the concentration of the antigen in each serum sample (*n* = 31) and urine sample (*n* = 42) sample from melioidosis patients (Table 1). The limits of detection (LODs) for the CPS antigen capture ELISA developed in our laboratory are approximately 0.0083 ng/mL in buffer, 0.010 ng/mL in serum, and 0.0033 ng/mL in urine (see Fig. S1 in the supplemental material). The results from assaying the serum samples showed that CPS was detected in 48.4% (15/31) of serum samples, with antigen concentrations ranging from 0.017 ng/mL to 210 ng/mL. Fourteen of the 15 patients with CPS detected in serum had results available from contemporaneously taken blood cultures, 9/14 (64%) of which grew B. pseudomallei. All of the 16 patients with CPS not detected in serum (i.e., either CPS negative or CPS present at a concentration below the LOD of the antigen capture ELISA) had contemporaneous blood culture results; 9/16 (56%) of these cultures grew B. pseudomallei. Analysis of neat urine resulted in the detection of CPS in 59.5% (25/42) of samples, with the antigen concentrations ranging from 0.017 ng/mL to 1,300 ng/mL. CPS was not detected in 40.5% (17/42) of neat urine samples.

**TABLE 1:** Sites of Burkholderia pseudomallei infection and corresponding CPS ELISA and AMD LFI results for serum and urine samples

Patient ID^*a*^	Disease category^*b*^	Presence of B. pseudomallei-positive blood culture	Other B. pseudomallei culture-positive sample(s)^*c*^	Serum concn by ELISA (ng/mL)^*d*^	LFI result for serum^*e*^	Urine concn by ELISA (ng/mL)^*d*^	LFI result for urine^*e*^
MM832	DISS, 2a	+	TS	<LOD	−	<LOD	−
MM834	DISS, 3abh	+		<LOD	+/−	<LOD	−
MM838	LOC, 5f	−	UR	0.33	+/−	<LOD	−
MM842-1	DISS, 4	−	UR, TS	<LOD	−	0.017	−
MM842-2	DISS, 4	−	UR, TS	NA	NA	0.036	−
MM844	DISS, 2c	+		<LOD	−	<LOD	−
MM850-1	DISS, 2d	+		0.17	−	540	+
MM850-2	DISS, 2d	+		NA	NA	1.9	+
MM857	DISS, 2b	+	P	<LOD	−	<LOD	−
MM859	DISS, 1	+		0.020	−	0.020	−
MM861	DISS, 3ah	+	TS, P	0.051	−	65	+
MM871	DISS, 2a	+	TS	<LOD	−	42	+
MM875	LOC, 5d	−	P	<LOD	+/−	<LOD	−
MM876	LOC, 5j	−	TS	<LOD	−	<LOD	−
MM878-1	DISS, 3af	+	UR	<LOD	−	180	+
MM878-2	DISS, 3af	+	UR	NA	NA	340	+
MM879	DISS, 3ah	+	TS, P	<LOD	−	<LOD	−
MM881	DISS, 2d	+	TS	<LOD	−	<LOD	−
MM882	LOC, 5d	−	TS, P	0.30	+	3.1	+
MM883	LOC, 5d	−	P	0.024	−	<LOD	−
MM884	DISS, 3af	+	UR, TS	0.66	+/−	0.81	+
MM885	DISS, 3bd	−	TS, P, SP	<LOD	−	0.073	+
MM889	DISS, 2d	+	P	NA	NA	<LOD	−
MM890	DISS, 2a	+	TS	0.018	−	<LOD	−
MM891-1	LOC, 5f	−	UR	0.077	+/−	15	+
MM891-2	LOC, 5f	−	UR	NA	NA	5.5	+
MM893	DISS, 2f	+	UR	<LOD	−	22	+
MM900	LOC, 5d	−	P	NA	NA	<LOD	−
MM901	LOC, 5d	−	P	0.021	+/−	<LOD	−
MM903	LOC, 5g	−	P	<LOD	−	<LOD	−
MM904	DISS, 2j	+	TS	0.017	−	0.035	−
MM905	DISS, 2a	+		210	+	1,300	+
MM906	LOC, 5i	−	P	NA	NA	0.059	−
MM909-1	DISS, 2j	+	TS	NA	NA	0.2	−
MM909-2	DISS, 2j	+	TS	NA	NA	0.092	−
MM912	LOC, 5j	−	TS	<LOD	−	<LOD	−
MM914-1	DISS, 3ah	+	TS, P	0.066	−	0.52	+
MM914-2	DISS, 3ah	+	TS, P	NA	NA	5.1	+
MM916	DISS, 4ad	−	P	<LOD	−	<LOD	−
MM919-1	DISS, 3dhf	+	UR, P	0.028	−	520	+
MM919-2	DISS, 3dhf	+	UR, P	NA	NA	490	+
UI33259	NA	NA	NA	0.030	−	0.062	+/−

a Patients with an initial identifier and then -1 or -2 are the same patient where multiple sample sets were collected during the same episode of illness.

b DISS, disseminated, where 1 indicates bacteremia only, 2 indicates bacteremia with a single focus, 3 indicates bacteremia with multiple foci, and 4 indicates negative blood cultures but multiple foci; LOC, localized, where 5 indicates a single focus (with negative blood cultures and no clinical or radiological evidence of additional foci). Foci (identified clinically plus a positive microbiological sample from that site and/or imaging identifying abscess formation at that site) are categorized as follows: a for lung infection, b for liver abscess, c for splenic abscess, d for skin and soft tissue infection, e for parotitis, f for urinary tract infection, g for lymphadenitis, h for bone and joint infection, i for tonsilitis, and j for unknown.

c UR, urine; TS, throat swab; P, pus; SP, sputum.

d NA denotes cases where (i) the test was not performed due to a limited sample volume (ELISA and LFI) or (ii) data were not available (disease category). <LOD, below the limit of detection.

e LFI results are reported as positive (+) or negative (−) when results were consistent among all three observers; +/− indicates cases where one or two observers reported the LFI as positive.

### Detection of CPS in patient samples by the AMD LFI.

**FIG 1:**
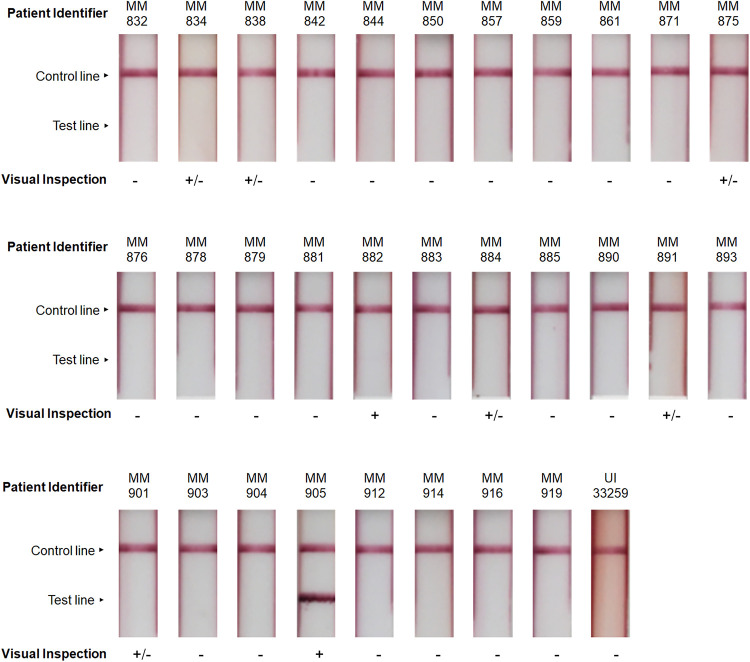
AMD LFI analysis of melioidosis patient serum samples. AMD LFIs tested with melioidosis patient serum samples were evaluated visually by three blinded individuals. Unanimous results for the visual readouts are reported as positive (+) or negative (−). Discordant results where at least one but not all individuals recorded the test as positive, are reported as (+/−).

Each of the 31 serum samples and 42 urine samples was tested by the AMD LFI. The RDTs were visually assessed for reactivity. For the serum samples, three individuals performing evaluations in a blind manner unanimously evaluated samples MM882 and MM905 as being positive (Table 1 and Fig. 1). Six serum samples (MM834, MM838, MM875, MM884, MM891, and MM901) had ambiguous results, indicated as “+/−,” where at least one individual called the test positive and at least one called the test negative. It was unanimously determined that the remaining 23 serum samples were negative.

**FIG 2:**
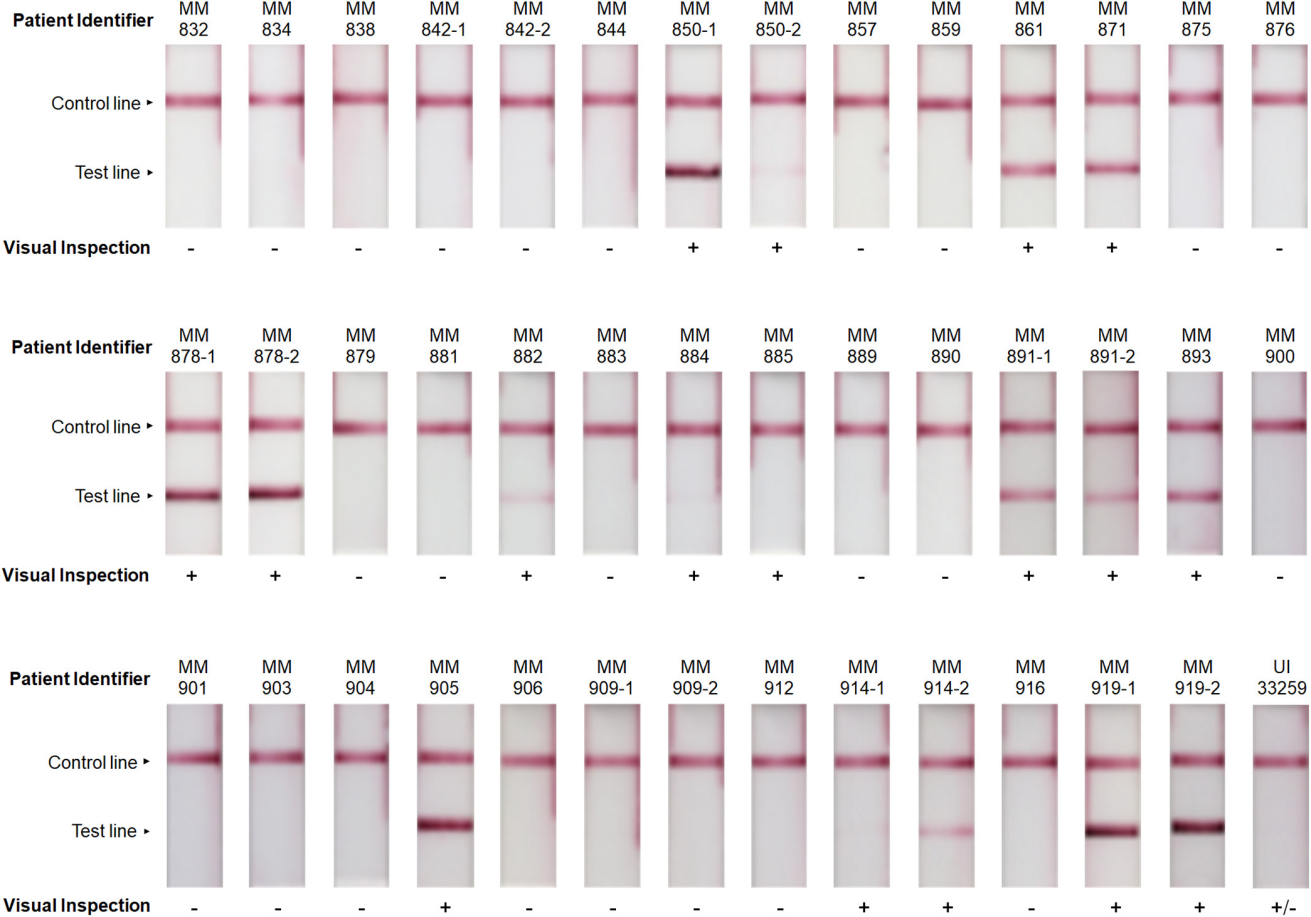
AMD LFI analysis of melioidosis patient urine samples. AMD LFIs tested with melioidosis patient urine samples were evaluated visually by three blinded individuals. Unanimous results for the visual readouts are reported as positive (+) or negative (−). Discordant results where at least one but not all individuals recorded the test as positive, are reported as (+/−).

Testing of urine samples showed that 17/42 samples were reported as being positive by the AMD LFIs by all three individuals (Table 1 and Fig. 2). One urine sample, from patient UI33259, the patient who did not have culture-confirmed melioidosis, was considered positive by one and negative by the other two individuals. Results were in agreement that the remaining 24 samples were negative by the RDT.

### Site of disease.

Melioidosis diagnosis was based on a positive culture result from blood, throat swabs, urine, pus, or sputum. The disease state was categorized as either disseminated or localized infection. Melioidosis cases were defined as “disseminated” when B. pseudomallei bacteremia was present and/or there was clinical, radiological, or microbiological evidence of multiple sites of disease. Blood, throat, and urine cultures were recommended for all patients whenever melioidosis was suspected, along with culture of pus from abscesses, body fluids, and sputum when clinically indicated. Cases were defined as “localized” if only one site of disease was present.

### Concentration of urine samples with low levels or no detectable CPS.

**FIG 3:**
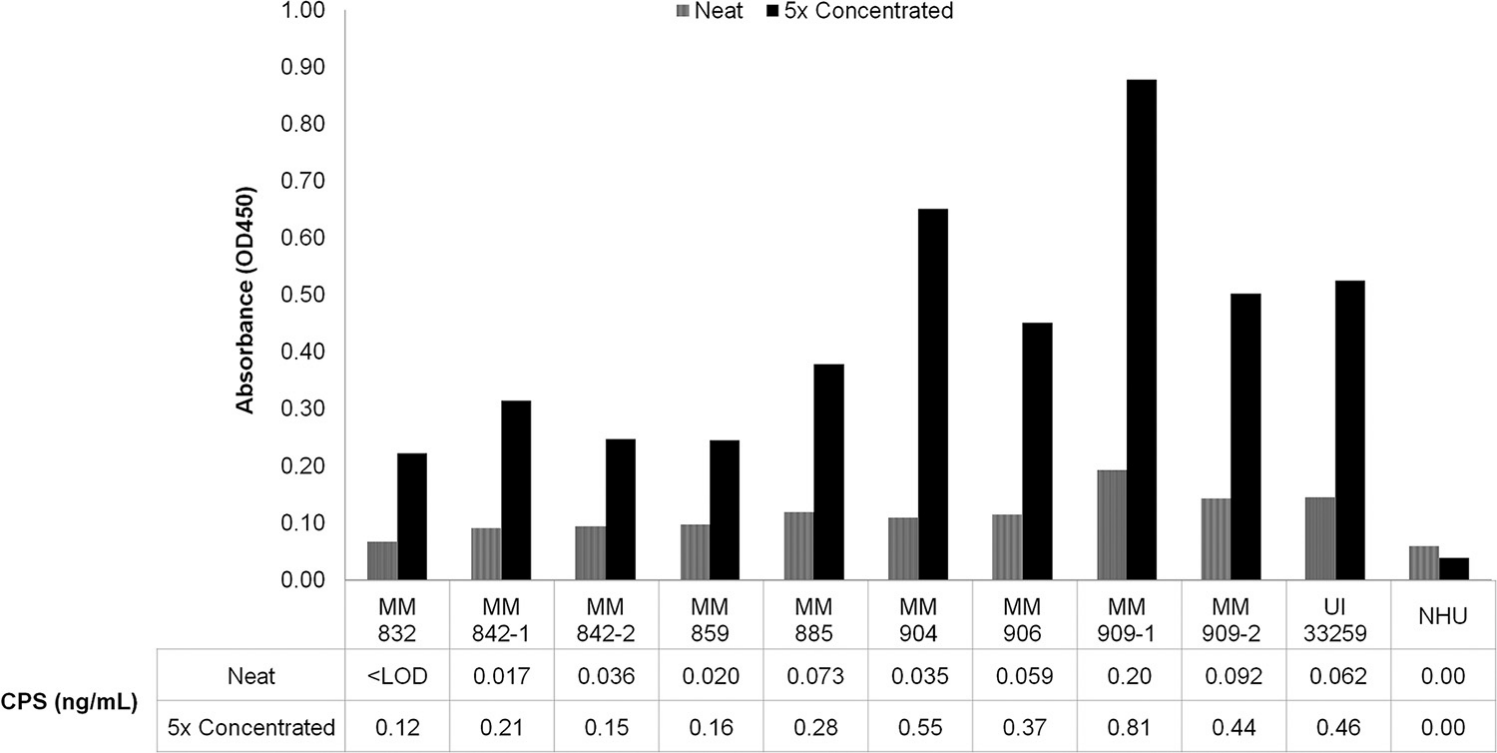
Antigen-capture ELISA analysis of melioidosis patient urine samples pre- and post-concentration. CPS antigen-capture ELISA results (OD_450_) and corresponding concentrations of CPS (ng/mL) for patient urine samples before and after a 5-fold concentration step. Samples selected for concentration showed either low or undetectable amounts of CPS after initial ELISA analysis. Samples that remained negative are not shown.

Eight out of 42 urine samples had CPS detectable by the ELISA (0.017 ng/mL to 0.092 ng/mL) but were either negative or yielded a weakly positive result by the AMD LFI. To determine whether a preconcentration step could serve to enhance CPS detection in urine, each of the 8 low-CPS-positive urine samples and 17 negative urine samples was concentrated 5-fold and evaluated again by the antigen capture ELISA. This method was employed with urine samples only, as the volume of serum available from these patients was more limited. Additionally, concentration of serum for this purpose is challenging given its greater viscosity than that of urine. Analysis of urine samples with initially low levels of CPS revealed that the 5-fold reduction in volume did increase the concentration of CPS in all samples (Fig. 3). After concentration, CPS was also detected in 1/17 initially negative samples (patient MM832). All other negative samples remained below the LOD of the ELISA and are not reported.

**FIG 4:**
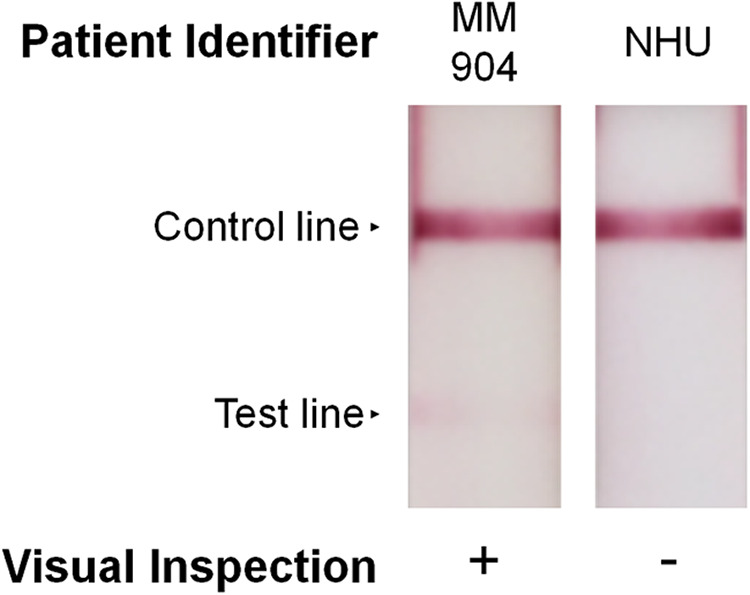
Concentrated MM904 detection on AMD LFI. Urine from MM904 was concentrated 5-fold and analyzed on the AMD LFI. A negative control of 5-fold concentrated normal human urine was evaluated alongside the concentrated sample.

For the majority of the urine samples that were concentrated, initial sample volumes limited testing to a single replicate by the antigen capture ELISA. There was, however, a sufficient volume of urine from patient MM904 to allow additional testing of the concentrated sample by the AMD LFI (Fig. 4). While the urine from patient MM904 was initially negative by the AMD LFI, three individuals performing evaluations in a blind manner unanimously assessed the concentrated sample to be positive by the RDT. As a negative control, concentrated normal human urine (NHU) was evaluated side by side using the AMD LFI.

In conclusion, the results from this study provide valuable data revealing the concentrations of the B. pseudomallei diagnostic antigen CPS in paired serum and urine samples from melioidosis patients. Data from both the laboratory-based ELISA and the point-of-care AMD LFI RDT support urine as a preferable sample matrix for the detection of CPS, in addition to pus, body fluids, or sputum where these are available. The detection of CPS in urine, by either an ELISA or AMD, should prompt the initiation of appropriate treatment and further investigation for disseminated foci of melioidosis (e.g., abdominal ultrasound to identify possible occult liver or spleen abscesses). The results from the study further indicate that sample concentration is a viable and promising method for improving the detection of CPS in urine.

## DISCUSSION

Their ease of use, rapid results, and affordability make RDTs powerful tools for the diagnosis of infectious diseases. The AMD LFI is capable of the quick and accurate identification of B. pseudomallei CPS for the diagnosis of melioidosis. While the assay is quite analytically sensitive, especially for an RDT, there are limited data to establish whether the assay meets the requisite analytical sensitivity for clinically relevant levels of CPS in serum and urine samples. In this study, the collection of paired serum and urine samples from Lao melioidosis patients enabled a detailed analysis of CPS detection in the two matrices. The major goals were to (i) investigate and compare the concentrations of CPS in serum and urine samples from melioidosis patients, (ii) evaluate AMD LFI results for the same samples, and (iii) examine whether sample concentration may be a viable method for improving detection in melioidosis samples with borderline or undetectable levels of CPS.

An antigen capture ELISA of CPS levels in serum and urine samples from melioidosis patients revealed that serum levels ranged from 0.017 ng/mL to 210 ng/mL in 15/31 samples and that the levels in urine ranged from 0.017 ng/mL to 1,300 ng/mL in 25/42 samples. The remaining samples were negative or below the LOD of the antigen capture ELISA. In addition to a higher percentage of urine samples being positive for CPS detection, the overall concentrations of CPS were higher in urine than in serum. For reference, the CPS concentration was above 1 ng/mL in more than half of the positive urine samples, while the concentrations in all but one of the positive serum samples were below 1 ng/mL. The detection of CPS in urine was more frequent with disseminated forms of the disease. Twenty-three of the 34 culture-confirmed patients were categorized as having disseminated disease (Table 1). CPS was detected in urine in 14/23 (60.9%) patients by the ELISA and in 10/23 (43.5%) by AMD. In comparison, only 3/11 (27%) patients with localized disease were positive in urine by the ELISA; 2/11 (18.2%) were positive by AMD, and both of these patients had evidence of an involvement of the urinary tract as a focus of infection.

The corresponding AMD LFI results further supported the use of urine for CPS detection, with more of the urine samples yielding a positive result (17/42) than serum samples (2/31). Multiple serum samples yielded results that were not definitively positive or negative. The individuals performing evaluations in a blind manner noted that when testing serum samples, the assays were more likely to have streaking, which added difficulty to the evaluation of faint test lines. Of the samples that were positive by the antigen capture ELISA, 10/15 serum samples and 9/25 urine samples had levels of CPS below the LOD of the AMD LFI (200 pg/mL). These data give further insight into the results of AMD LFIs and the associated challenge of detecting an antigen that, at times, can be circulating at very low levels. Many of the samples that had CPS levels below 200 pg/mL were, in fact, negative by the AMD LFI. However, there were exceptions for both serum (MM838, MM875, MM891, and MM901) and urine (MM885 and UI33259) samples, where these samples yielded either a positive or an ambiguous (+/−) result. Alternatively, a serum sample (MM884) with a CPS concentration (660 pg/mL) well above the LOD of the AMD LFI did not yield an AMD result that was clearly positive to those evaluating the assays. When assessing these results, it is important to note that LODs are typically established using normal human matrices spiked with antigen to provide an estimate of assay sensitivity. However, when testing clinical samples from ill patients, there are factors that can interfere with antigen detection at the test line. Melioidosis patients often present with renal failure, diabetes mellitus, and liver cirrhosis, which may affect the levels of glucose, protein, blood, and pH of patient serum and urine samples (19–23). These substances, as well as others, are known to alter the signal strength of immunoassays (24–26).

It should also be noted that the 73 samples in this study were processed through a 0.2-μm filter before testing for safety reasons. As such, any bacteria in the samples were removed prior to analysis. The concentration of B. pseudomallei in culture-positive patient serum samples tends to be low, with a documented median concentration of 1.1 CFU/mL (9, 10). In a study performed previously by Wuthiekanun et al., the median concentration of B. pseudomallei in culture-positive urine samples was 1.5 × 10^4^ CFU/mL and could be ≥1 × 10^6^ CFU/mL (10). Despite the fact that a larger number of bacteria are present in urine when cultures are positive, the same study also showed that, overall, only 21% of urine cultures were positive in patients with confirmed melioidosis, compared with 49% of blood cultures. Although the sensitivity of urine culture for the diagnosis of melioidosis is relatively low, a study using purified CPS in a mouse model indicated that molecules of the circulating CPS antigen are quickly filtered out of the blood through the kidneys and are detectable in urine (18). As CPS is both shed and cell associated, it is possible that testing unfiltered samples might show higher concentrations of CPS and a higher rate of positive AMD LFI results (27, 28).

CPS was detected in 59.5% of urine samples and 48.3% of paired serum samples by the antigen capture ELISA. Comparatively, CPS was definitively detected in 40.5% of urine samples and 6.5% of serum samples by the AMD LFI. The results from this study indicate that higher levels of CPS are found in urine than in serum. Urine samples that either were negative or showed low levels of CPS by the antigen capture ELISA presented an opportunity to further study whether sample concentration could increase the relative concentration of CPS prior to diagnostic testing. Importantly, the ability to collect larger sample volumes of the intended matrices is an essential requirement for this approach. Given that CPS has been shown to be detectable in urine from some melioidosis patients, this matrix has an additional advantage over serum as it is readily available in large quantities using noninvasive techniques from most patients. In this study, the available volume of urine from most patients allowed the testing of a single replicate by the antigen capture ELISA after 5-fold concentration via a centrifugal spin concentrator. The results of this testing are promising, as the data show that postconcentration levels of CPS were increased for all of the low-CPS samples. Additionally, CPS became detectable in one sample (MM832) that was initially negative by the quantitative immunoassay. This amplification of the signal by centrifugal concentration of urine might be worthwhile for patients strongly suspected of having melioidosis, for both negative samples and those with faint bands that can be perceived as ambiguous results.

In order to evaluate the applicability of this approach to real-world clinical testing, excess concentrated sample from patient MM904 was evaluated by the AMD LFI and compared to the testing of a neat sample. The shift from a negative to a positive result after sample concentration demonstrates the potential for improving the detection of CPS and the subsequent diagnosis of melioidosis. A valid concern regarding this methodology is that some point-of-care testing sites in areas of endemicity may not have the infrastructure or funding to support sample concentration using a centrifuge. Recent advances in gravity-driven, static concentration technologies (i.e., Sartorius Vivapore concentrators) are providing exciting alternatives to traditional techniques by enabling straightforward sample concentration that can be performed in resource-limited point-of-care settings.

## MATERIALS AND METHODS

### Patient samples.

Archived paired serum and urine samples from patients with confirmed or suspected melioidosis were obtained from the Lao-Oxford-Mahosot Hospital Wellcome Trust Research Unit (LOMWRU). Ethical approval was obtained from the Oxford Tropical Research Ethics Committee and the Lao National Ethics Committee for Health Research. All adult patients gave written informed consent for participation, and written consent was obtained from the parent/guardian of each participant under 16 years of age. Patients were confirmed to be positive for melioidosis by culturing blood, throat swabs, urine, pus, or sputum. Patients were treated according to international recommendations, usually with ceftazidime during the intensive phase and co-trimoxazole during the eradication phase, once the diagnosis of melioidosis had been confirmed (29). Samples for culture were processed in real time as previously described (15). Serum was separated by centrifugation and aliquoted, and both serum and urine samples were then stored at −80°C before shipment to the United States on dry ice. Upon receipt at the University of Nevada, Reno, samples were again stored at −80°C. Prior to the ELISA and LFI, samples were thawed and 0.2-μm sterile filtered in a biosafety level 3 laboratory. Each sample was verified for sterility by back-culturing and then transferred to a biosafety level 2 laboratory for subsequent analyses. Patient serum and urine samples with matching patient identifiers were taken on the same collection day. Seven patients had two urine samples collected during the course of the same episode of illness. Both samples for these individuals were included in the analysis, as indicated by the -1 or -2 following the patient identifier in Table 1.

### CPS quantitative antigen capture ELISA.

An antigen capture ELISA was performed as previously described, using the monoclonal antibody (mAb) 4C4 that is reactive to B. pseudomallei CPS (18, 30). Briefly, 96-well plates were coated with 100 μL/well of 2.5 μg/mL of mAb 4C4 diluted in phosphate-buffered saline (PBS) and incubated overnight. Wells were washed and blocked. Samples were diluted to 1 part sample with 1 part blocking solution (phosphate-buffered saline with 5% skim milk and 0.5% Tween 20) for a 2-fold dilution initially unless otherwise mentioned. Samples were then 2-fold serially diluted across the plate for a final volume of 100 μL/well and incubated for 90 min. A standard curve was generated with purified B. pseudomallei CPS (courtesy of Paul Brett and Mary Burtnick, Department of Microbiology and Immunology, University of Nevada, Reno, School of Medicine) at a starting concentration of 30 ng/mL. Purified CPS was spiked into normal human serum (BioIVT, Westbury, NY) or normal human urine (Innovative Research, Inc., Novi, MI) and used to assess the concentrations of CPS in patient serum or urine samples, respectively. Wells were again washed and incubated with horseradish peroxidase (HRP)-conjugated mAb 4C4 at 0.5 μg/mL for 60 min (100 μL/well). HRP conjugation was performed using EZ-Link Plus activated peroxidase (Thermo Fisher Scientific, Waltham, MA). After washing again, wells were incubated with the tetramethylbenzidine substrate for 30 min (100 μL/well) (SeraCare, Milford, MA), followed by the addition of 1 M H_3_PO_4_ (100 μL/well), and the optical density at 450 nm (OD_450_) was then read. Each sample was analyzed in triplicate unless otherwise indicated.

### Concentration of urine samples.

Patient urine samples that were negative or had CPS concentrations near the limit of detection (LOD) in the antigen capture ELISA were concentrated 5-fold using Amicon Ultra centrifugal filters (3,000-molecular-weight cutoff [MWCO]; MilliporeSigma, Darmstadt, Germany). The concentrated urine samples were then analyzed by a quantitative antigen capture ELISA as described above. The normal human urine used for the calibration curve was also concentrated 5-fold prior to the addition of the CPS standard. Patient samples were analyzed as a single replicate due to sample volume limitations.

### Active Melioidosis Detect rapid test lateral flow immunoassay.

The RDTs were performed according to the manufacturer’s recommendations and were performed at room temperature. When testing patient serum samples, 35 μL of serum was placed on the test strip. The strip was then inserted into a 96-well microtiter plate containing 3 drops (~150 μL) of chase buffer and allowed to run for 15 min. When testing patient urine samples, 50 μL of urine was mixed with 2 drops (~100 μL) of chase buffer in a 96-well microtiter plate. The test strip was then inserted into the well and allowed to run for 15 min. Each test strip includes a control line that turns red to indicate a valid test. The control line must be visible for the test to be further evaluated for the presence or absence of a visible test line. A positive test result is indicated by a red test line and indicates the presence of CPS. Test strips were visually evaluated as positive or negative by three readers who determined and recorded their individual results independently in a blind manner.
