# Supplementary material for: Detection and Quantification of the Capsular Polysaccharide of Burkholderia pseudomallei in Serum and Urine Samples from Melioidosis Patients
Source: Microbiol Spectr. 2022 Aug 4;10(4):e00765-22. doi: 10.1128/spectrum.00765-22 (PMC9430648; doi:10.1128/spectrum.00765-22)
Supplement: Supplemental file 1 — Supplemental material. Download spectrum.00765-22-s0001.pdf, PDF file, 0.1 MB [file spectrum.00765-22-s0001.pdf]

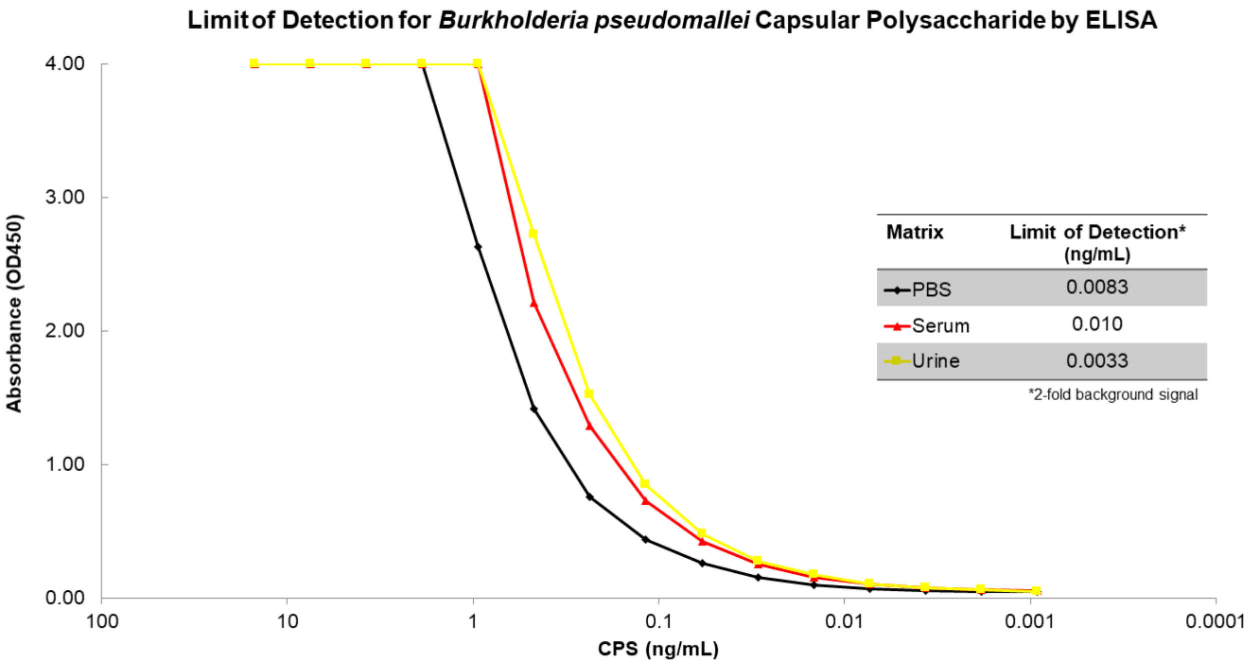

FIG S1 Standard curves of the antigen-capture ELISA using purified CPS spiked in PBS buffer, normal human serum and normal human urine. Plots represent an average of into two independent lots of pooled serum or urine, run in triplicate. The limit of detection is defined as 2-fold background signal.
